# Thin Porous Poly(ionic liquid) Coatings for Enhanced Headspace Solid Phase Microextraction

**DOI:** 10.3390/polym12091909

**Published:** 2020-08-24

**Authors:** David J. S. Patinha, Hong Wang, Jiayin Yuan, Sílvia M. Rocha, Armando J. D. Silvestre, Isabel M. Marrucho

**Affiliations:** 1 Instituto de Tecnologia Química e Biológica António Xavier, Universidade Nova de Lisboa, Av. Da República, 2780-157 Oeiras, Portugal; davidpatinha@gmail.com; 2 CICECO—Aveiro Institute of Materials and Department of Chemistry, University of Aveiro, 3810-193 Aveiro, Portugal; armsil@ua.pt; 3 Key Laboratory of Functional Polymer Materials, Ministry of Education, Institute of Polymer Chemistry, Nankai University, Tianjin 300071, China; hongwang1104@nankai.edu.cn; 4 Department of Materials and Environmental Chemistry, Stockholm University, 10691 Stockholm, Sweden; 5 LAQV-REQUIMTE & Department of Chemistry, University of Aveiro, Campus Universitário de Santiago, 3810-193 Aveiro, Portugal; smrocha@ua.pt; 6 Centro de Química Estrutural and Departamento de Engenharia Química, Instituto Superior Técnico, Universidade de Lisboa, Avenida Rovisco Pais, 1049-001 Lisboa, Portugal

**Keywords:** solid phase microextraction (SPME), poly(ionic liquid), interpolyelectrolyte complexation, porous materials, surface coating

## Abstract

In this contribution, thin poly(ionic liquid) (PIL) coatings with a well-defined pore structure built up from interpolyelectrolyte complexation between a PIL and poly(acrylic acid) (PAA) were successfully used for enhanced solid phase microextraction (SPME). The introduction of porosity with tunable polarity through the highly versatile PIL chemistry clearly boosts the potential of SPME in the detection of compounds at rather low concentrations. This work will inspire researchers to further explore the potential of porous poly(ionic liquid) materials in sensing and separation applications.

## 1. Introduction

Solid phase microextraction (SPME) is the most used microextraction technique at present and was initially introduced as early as the 1990s by Pawliszyn et al. [1]. As SPME combines the sample preparation and sampling together in a single step, it became one of the most popular, frequently investigated pre-concentration techniques for the detection and analysis of volatile organic compounds (VOC) that have compounding long-term health effects, but their low concentrations and the slow-developing symptoms have restricted VOC research [2,3,4]. In addition, the sample preparation procedure in SPME is easy to implement, non-invasive/non-destructive, and robust. Therefore, SPME is becoming more and more popular in a broad range of fields, including, but not limited to, environment science [5], food chemistry [6], and in in vivo studies [7]. Mechanistically, the SPME technique relies on the partition of analyte molecules between the sample matrix and the extraction phase, either in direct immersion (DI) or headspace (HS) extraction modes. In this context, the choice and design of the extraction phase is crucial in determining SPME efficiency. Usually, gas chromatography is the choice of detection method, with the SPME fiber (containing the extraction phase) inserted in the injector port to desorb analyte molecules, where GC programs with high temperatures ranging from 200 °C to 300 °C are preferred, to accelerate the release and detection of the analytes. Among various geometries, the fiber geometry was the first to be proposed [1], which remains the most popular to date [8]. The state-of-the-art extraction phases are dense polymeric fibers, such as poly(dimethylsiloxane) to detect non-polar samples, polyacrylate for polar samples, and the mixtures of polydivinylbenzene, poly(dimethylsiloxane), and carboxen for a broader range of analytes in complex samples [9,10]. To date, the available fibers for SPME in the market still suffer from some limitations, such as the low chemical and thermal stabilities, resulting in loss of reproducibility, excessive swelling in organic solvents, fragility, poor selectivity and/or low extraction efficiency [11,12]. To counteract such dilemma, the search for innovative materials as improved extraction phases is an active topic of utmost importance in the SPME field.

Poly(ionic liquid)s are innovative polyelectrolytes carrying ionic liquid species in their repeating units, and are typically produced by polymerization of ionic liquid monomers, or by engineering ionic liquid species covalently into the polymer repeating units [13,14]. Such structure synergy, i.e., polymer chains plus the ionic liquid species, allows for the combination of classic properties of polymers with that of ionic liquids, creating a vast group of functional task-specific polymer materials [15]. PILs in SPME application were first introduced by Anderson in 2008 [16], and thereafter have been explored for the extraction of a wide range of analytes, using the fiber coatings obtained mainly by dip-coating into a PIL solution. Other methodologies such as electropolymerization, silica-mold, and spray-coating have also been tested [17,18,19]. Nonetheless, despite much success as new easy-tunable materials [20], the slow kinetics in the extraction process, i.e., the enrichment of analyses from the sampling sites at ambient or low temperatures, can potentially hamper their full potential [21,22,23,24,25,26,27,28]. In this context, porous SPME matrices can be key to overcome this issue. The development of porous poly(ionic liquid) (PIL) materials currently attracts much interest, as they combine high surface area of porous materials and flexible structure design in polymers with the unique physicochemical properties of ionic liquids, resulting in materials with tunable surface area and pore structures, mechanical and thermal stability, chemical architectures, and possible host–guest interaction [29]. Due to the increase in surface exposure afforded by the porous structure that enhances interfacial mass and energy exchange, these materials find a wide range of applications in sensing, separation, electronic devices and catalysis.

In this work, a facile and straightforward strategy based on the electrostatic complexation method is used to create an innovative porous PIL SPME (pPIL-SPME) coating, built up from a PIL and PAA. The introduction of porosity with tunable polarity through the highly versatile PIL chemistry clearly boosts the potential of SPME in the detection of compounds of low concentration.

## 2. Materials and Methods

### 2.1. Materials and Instruments

#### 2.1.1. Chemicals

Vinylimidazole (99%), bromoacetonitrile (97%), poly(acrylic acid) (PAA) (*M_w_*—2000 Da), and poly(N,N-dimethyldiallylammonium chloride) (*M_w_*—400,000–500,000 Da) were purchased from Sigma Aldrich (Gillingham, United Kingdom). Lithium bis(trifluromethane sulfonyl)imide (LiTFSI, 99.95%) was purchased from Io-li-tec (Heilbronn, Germany). All chemicals were used without any further purification. Solvents were of analytical grade. Pentanoic acid (>99%), hexanoic acid (>99%), 3-pentanone (>99%), 2-hexanone (>98%), 2-heptanone (>98%), 2-methylbutanal (98%), 1-heptanal (95%), 1-hexanol (99.9%), 2–heptanol (98%), 1–octanol (99.7%), benzyl alcohol (99.8%), 1-ethylnaphtalene (>97%), carvacrol (>98%), (+)-α-pinene (>99%), α-terpineol (>99%), eucalyptol (99%), (R)-(+)-pulegone (97%), and β-citronellol (95%) were purchased from Sigma-Aldrich (Gillingham, United Kingdom). BTEX: Benzene (B) (99%) was provided by Riedel-de Haen, (Charlotte, NC, USA). Toluene (T) (99%), ethylbenzene (E) (99%), and p-xylene (X) (99%) were obtained from Merck (Darmstadt, Germany).

#### 2.1.2. Instruments

Nuclear magnetic resonance spectroscopy (NMR): ^1^H-NMR spectra were recorded at room temperature using a Bruker (Billerica, MA, USA), DPX-400 spectrometer operating at 400 MHz. Deuterated dimethylsufoxide (DMSO-*d_6_*) was used as a solvent for the measurement.

Scanning electron microscopy (SEM): The morphology of the samples was studied by scanning electron microscopy conducted on a JEOL 7000 (Tokyo, Japan), operated at 3 kV. Samples were coated with a thin gold layer for 40 s before examination.

Fourier Transform-Infrared Spectroscopy (FT-IR): The FT-IR spectra of the samples were performed on a BioRad 6000 FT-IR spectrometer (Hercules, CA, USA). Samples were measured in solid state using a Single Reflection Diamond ATR (attenuated total reflection).

Gel permeation chromatography (GPC) was performed using a NOVEMA (Mainz, Germany) Max linear XL column with a mixture of 80% of aqueous acetate buffer and 20% of methanol. Conditions: flow rate 1.00 mL min^−1^, PSS standards using RI detector-Optilab-DSP-Interferometric Refractometer (Wyatt-Technology, Dernbach, Germany).

Thermogravimetric analysis (TGA) experiments were conducted on a Netzsch (Selb, Germany), STA 449 F1 Jupiter^®^ apparatus. The experiments were performed under a heating rate of 10 K min^−1^ with a constant nitrogen flow.

The GC × GC–ToFMS system: a LECO Pegasus 4D (LECO, St. Joseph, MI, USA), containing an Agilent GC 7890A gas chromatograph (Agilent Technologies, Inc., Wilmington, NC, USA), incorporating a dual stage jet cryogenic modulator (licensed from Zoex), a secondary oven, and a mass spectrometer supplied with a ToF analyser. Analytes and chromatographic conditions can be found in Section 2.2.2 and Section 2.2.3.

### 2.2. Experimental Methods

#### 2.2.1. Synthesis of Poly(1-Cyanomethyl-3-Vinylimidazolium Bis(Trifluoromethane Sulfonyl)imide) (PCMVImTFSI)

**Figure 1:**
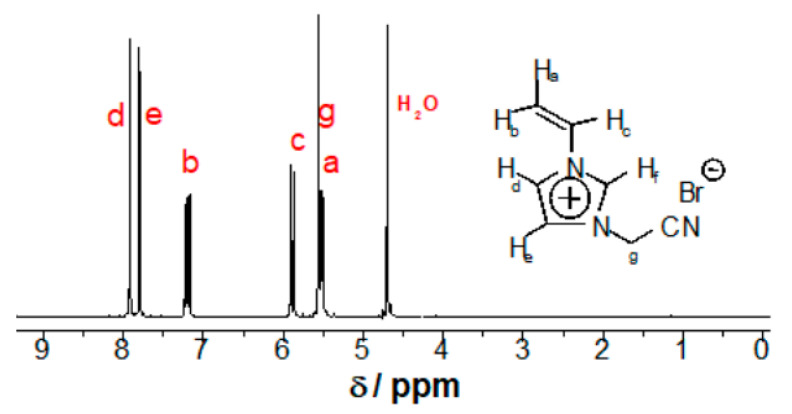
^1^H-NMR spectrum of 1-vinyl-3-cyanomethylimidazolium bromide in D_2_O.

The synthesis of the monomer and PIL follows a similar method reported previously [30,31]. 1-Vinyl-3-cyanomethylimidazolium bromide was produced by mixing 0.06 mol of 1-vinylimidazole with 0.06 mol of bromoacetonitrile in 20 mL of acetonitrile at 45 °C for 24 h with constant and vigorous stirring. The powder product was washed with ethyl acetate and its purity was proven by means of ^1^H NMR in Figure 1. Monomer 1-vinyl-3-cyanomethylimidazolium bromide (^1^H NMR (DMSO-*d_6_*, δ (ppm): 10.15 (s, 1H), 8.60 (t, 1H), 8.25 (t, 1H), 7.55 (dd, 1H), 6.15 (dd, 1H), 6.00 (s, 2H), 5.50(dd, 1H)).

**Figure 2:**
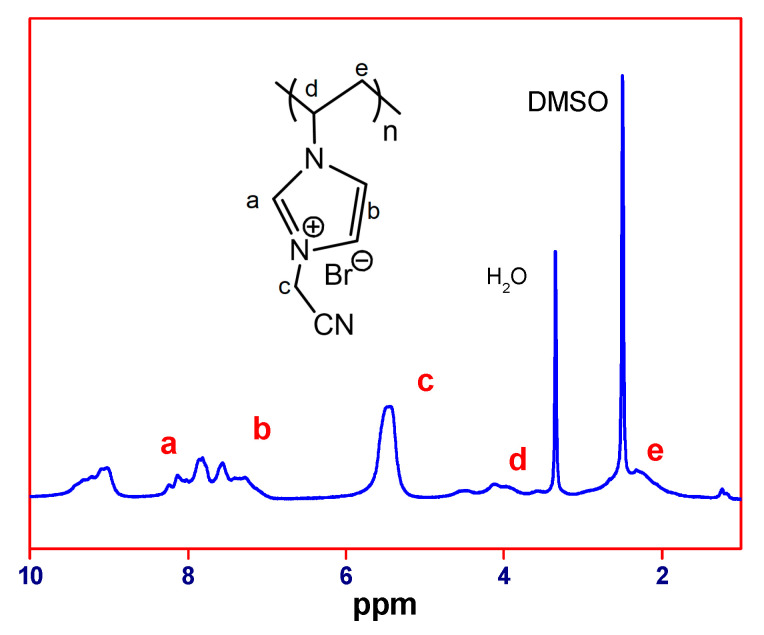
^1^H-NMR spectrum of PCMVimBr in DMSO-d*_6_*.

For the polymerization step, 10.38 g of the as-synthesized IL monomer (48.7 mmol), 30 mg of AIBN (0.175 mmol), and 70 mL of ethanol were loaded into a 250 mL reactor. The reactor was placed in an oil bath at 70 °C for 24 h, yielding poly(1-vinyl-3-cyanomethylimidazolium bromide) (PCMVImBr). The polymerization was followed by ^1^H NMR and, in the end, the resonances assigned to the double bond protons at *δ* = 6.15, 5.50, and 7.55 ppm disappeared (Figure 2). The polymer product in a powder form was obtained by precipitating the polymerization mixture in THF. The ^1^H-NMR of PCMVimBr was recorded in DMSO-d*_6_*. δ (ppm): 9.25 (1H), 8.20-7.10 (2H), 5.5 (2H), 4.70-3.80 (1H), 3.15-1.80 (2H),) (Figure 2).

Since water soluble PILs, such as PCMVImBr, do not yield porous materials using the proposed ionic complexation methodology, the bromide anions were exchanged with [TFSI^-^] anions. In a typical anion exchange, PCMVImBr was dissolved in water at a concentration of 1 wt.% and an aqueous solution of lithium bis(trifluoromethane sulfonyl)imide (LiTFSI) (TFSI/Br molar ratio is 1.15:1) was added dropwise, resulting in the precipitation of poly(1-vinyl-3-cyanomethylimidazolium bis(trifluoromethane sulfonyl)imide) (PCMVImTFSI) that was dried at 70 °C under vacuum until constant weight. The similar anion exchange procedure was also conducted to convert poly(*N,N*-dimethyldiallylammonium chloride) into poly(*N,N*-dimethyldiallylammonium TFSI).

#### 2.2.2. Preparation and Characterization of the Porous material.

As for the preparation of the pPIL-SPME fiber coating, 1.0 g of the synthetized PCMVImTFSI and 0.18 g of poly(acrylic acid) (PAA) were carefully dissolved in 10 mL of *N,N*-dimethylformamide until a homogeneous solution was obtained, and further stirred for 1 h. The pre-cleaned stainless steel wire was dipped in this solution for 10 min; after slowly withdrawing and drying the stainless steel wire, it was transferred to a 0.2 wt.% aqueous ammonia solution and left soaking for 4h to develop the porous polymer coating on the stainless steel wire surface. The resulting pore size distribution histogram shown was obtained by performing the measurement of 230 pores in their SEM images.

#### 2.2.3. Analytes

Ethanolic stock standard solutions composed of 22 compounds: pentanoic acid (Pent. Acid), hexanoic acid (Hex. Acid), 3-pentanone (3-Pen), 2-hexanone (2-Hex), 2-heptanone (2-Hep), 2-methylbutanal (Methyl But.), 1-heptanal, 1-hexanol, 2-heptanol, 1-octanol, benzyl alcohol (Bz. Alcohol), 1-ethylnaphtalene (Et. Nap.), carvacrol, (+)-α-pinene, α-terpineol, eucalyptol, (R)-(+)-pulegone, β-citronellol, benzene, ethylbenzene (Et. Benzene), toluene and p-xylene), were prepared at a concentration of 20 µg∙ml^−1^ of each analyte.

#### 2.2.4. Chromatographic Conditions

In the first dimension, an Equity-5 column (30 m × 0.32 mm I.D., 0.25 µm film thickness) was used, whereas a DB-FFAP column (0.79 m × 0.25 mm I.D., 0.25 µm film thickness) was used in the second dimension. The injector was maintained at 220 °C, using a splitless mode (30 s). The primary oven temperature program was: initial temperature 35 °C (hold 1 min), to 150 °C at 5 °C∙min^−1^ (hold 1 min) and then to 215 °C at 30 °C min^−1^ (hold 2 min). The secondary oven temperature program was 15 °C offset above the primary oven. The MS transfer line and MS source temperatures were 250 °C. The modulation time was 5 s (with hot and cold pulses through periods of 0.80 and 1.70 s, respectively); the modulator temperature was kept at 20 °C offset (above primary oven). Time of flight – mass spectrometer (ToF-MS) was operated at a spectrum storage rate of 85 spectra/s, in the EI mode at −70 eV using a range of *m/z* 30–350 and a detector voltage of 1638 V. Total ion chromatograms (TIC) were processed using the automated data processing ChromaTOF^®^ software (St. Joseph, MI., USA) at a signal-to-noise threshold of 100, and the DTIC (Deconvoluted Total Ion Current) GC × GC area data were used as an approach to estimate the relative content of each analyte, which were expressed as arbitrary units.

## 3. Results and Discussions

**Figure 3:**
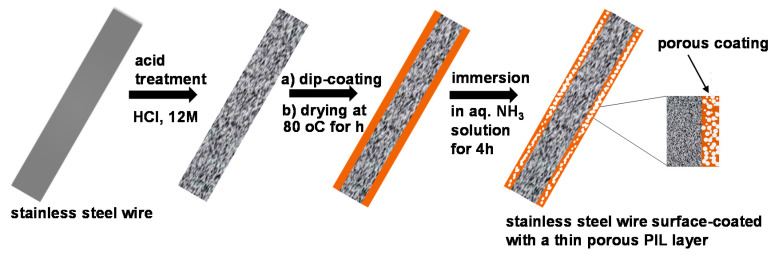
Scheme of the preparation of a porous PIL SPME (pPIL-SPME) fiber by coating a porous PIL thin film onto a stainless-steel wire surface.

Porous coatings using conventional polymers are usually prepared in the literature through a variety of techniques, such as polymer particle templating, in-situ polymerization, breath-figure approach, and block copolymer self-assembly [32,33,34,35]. In this work, the porous PIL SPME (pPIL-SPME) coating is produced via interpolyelectrolyte complexation between the hydrophobic PIL PCMVImTFSI and a weak polyelectrolyte PAA, a method that was locally invented by our group [30,31]. This method is very efficient in producing porous coatings, as it combines the pore formation process with the high affinity of PILs towards a variety of surfaces, particularly that of metals [15]. Figure 3 illustrates the preparation procedure of the pPIL-SPME fiber coating.

In detail, a hydrophobic imidazolium-type PIL, PCMVImTFSI, was used to prepare the pPIL-SPME coating due to its ionic and simultaneously hydrophobic nature. The successful synthesis of its monomer and polymer were verified by ^1^H-NMR spectra, shown in Figure 1 and Figure 2, where all chemical shifts can be assigned to individual protons in their corresponding chemical structures. First, a stainless steel wire of 220 ± 30 μm was chosen as the fiber substrate. After being etched by aqueous HCl of 12 M in concentration for 20 min, the stainless steel wire was vertically immersed for 1 h in a *N,N*-dimethylformamide (DMF) solution of a mixture of PAA and PCMVImTFSI, in a 1:1 equal molar ratio of the two repeating units. In addition, 5 wt.% (with regard to the PIL weight) of high molecular weight poly(dimethyldiallylammonium TFSI) obtained through anion metathesis reaction was also added to aid the film formation. Afterwards, the stainless-steel wire was carefully removed from the polymer mixture solution and placed vertically in an oven to be dried at 80 °C for 1h, to evaporate most of the DMF solvent. For the pore formation, the fibers with the polymer film coating were transferred to a 0.2 wt.% aqueous ammonia solution and left soaking for 4 h. This ammonia treatment resulted in a simultaneously evolved crosslinked porous polymer network, where the phase separation and the ionic complexation went hand-in-hand along with the diffusion of aqueous NH_3_ solution in the polymer film. The phase separation results from the diffusion of water molecules into the polymer thin coating, where the hydrophobic PCMVImTFSI adjusts itself to minimize its contact with water molecules. Meanwhile, the ionic complexation results from the concurrent diffusion of ammonia molecules into the polymer thin coating, which first neutralizes PAA into a polyanion poly(ammonium acrylate), that then complexes immediately with the surrounding polycation PCMVImTFSI.

**Figure 4:**
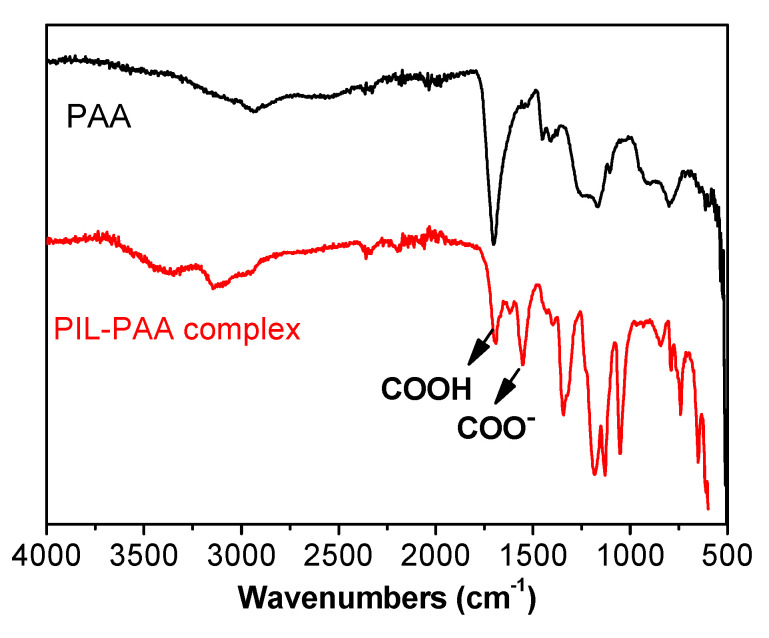
FT-IR spectra of PAA and the PCMVImTFSI-PAA complex in the porous coating film.

The neutralization of PAA in the polymer thin coating can be proven by the FTIR spectra of the original PAA and the as-prepared porous coating, as shown in Figure 4. The carbonyl band of the COOH groups at 1710 cm^−1^ in the pristine PAA splits into two bands in the crosslinked porous coating, one in its original position and the other at 1550 cm^−1^, which was ascribed to the C=O stretching in COO^-^ groups. The significant shrinkage of the COOH band and the appearance of an intense COO^-^ band in the coating product confirmed the deprotonation state of PAA. The phase separation and ionic complexation processes finish when water and ammonia molecules fully penetrate through the polymer thin coating, introducing pores into the crosslinked ionic networks.

**Figure 5:**
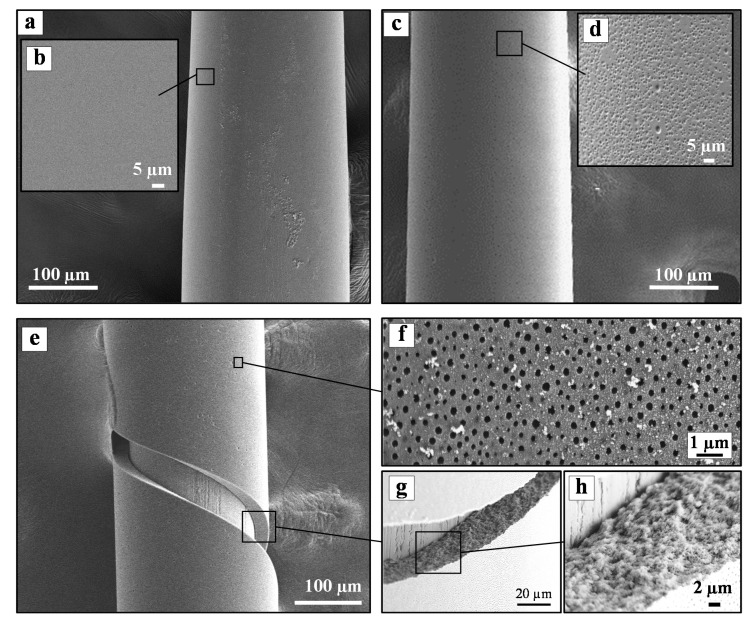
Images of the as-prepared PCMVImTFSI/PAA coatings; (**a**,**b**) no aqueous NH_3_ treatment; (**c**,**d**) after 1 h of aqueous NH_3_ treatment; and (**e**–**h**) after 4 h of aqueous NH_3_ treatment.

**Figure 6:**
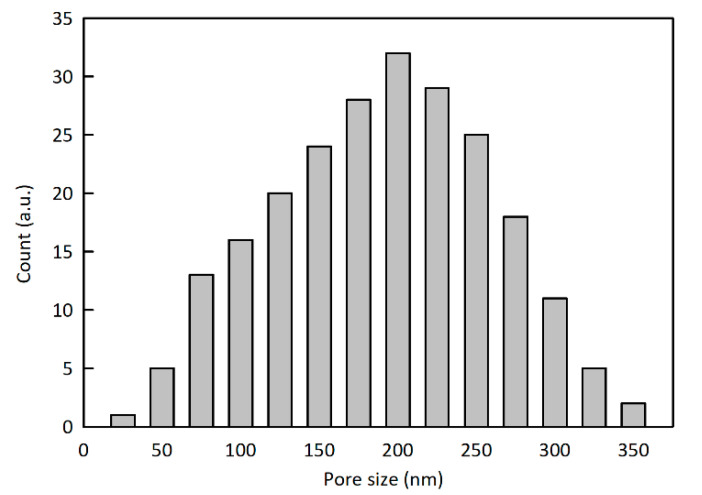
Size distribution histogram of 230 pores in the cross-section of the porous polymer coating, as measured by SEM from a sample obtained after 4 h aqueous NH_3_ treatment.

The scanning electron microscopy (SEM) images of the as-prepared nonporous (before immersion into aqueous ammonia solution) and porous PIL-SPME (after immersion into aqueous ammonia solution) fibers are shown in Figure 5. As can be seen, the coatings without aqueous ammonia treatment present a microscopically smooth dense surface without any visible pores (Figure 5a,b). The sample after 1 h immersion in an aqueous ammonia solution started to promote the formation of pores, and the surface is full of holes that have not yet penetrated inside the film, as indicated in Figure 5c,d. This immersion period of 1 h was further extended to 4 h to fully create the target porous structure inside the polymer coating. As can be observed, in the coating film surface, there are dense submicron pores across the entire surface, as shown in Figure 5f. In the cross-section area, a porous network structure was observed throughout the entire thickness of the fiber (Figure 5g–i). Both mesopores and macropores of diameters ranging from 25 nm to 350 nm are observed. Statistical pore size analysis shows an average size of 178 ± 37 nm (Figure 6). The average thickness of the porous coating is determined to be 20 ± 3 µm.

**Figure 7:**
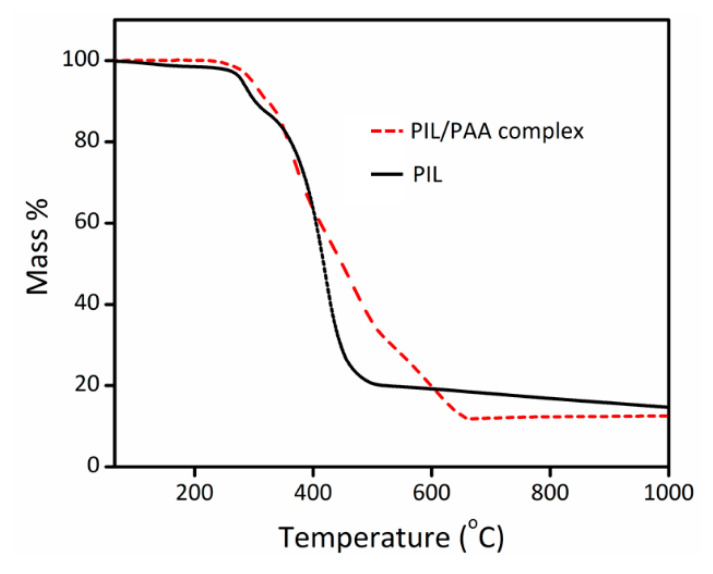
Thermogravimetric analysis (TGA) of the pristine PIL and the ammonia-treated pPIL-SPME coating film built up via interpolyelectrolyte complexation between PIL and PAA.

After successfully coating a porous ionic network film onto the stainless-steel wire, the thermal stability of this porous coating in terms of mass loss was evaluated in order to determine the proper heating program for the GC instrument. The thermogravimetric analysis of the polymer coating materials before and after pore formation shows the similar thermal stability, decomposing at > 260 °C (5% weight loss) (Figure 7). Therefore, the material is suitable for the application herein presented using a desorption temperature of max. 220 °C at the GC injection port. To note: the porous morphology of the polymer coating is also thermally stable and in fact can even be transferred into their porous carbon form at 1000 °C [36].

In order to test the prepared pPIL-SPME fibers, an ethanolic stock standard solution composed of 22 compounds was prepared at a concentration of 20 µg∙ml^−1^ of each analyte. A series of headspace-SPME (HS-SPME) triplicate extractions was carried during 30 min at 40 °C, with 50 µL of standard solution in 5 mL glass vials capped with a PTFE septum (Chromacol Ltd., Herts, UK). After each desorption, a fiber cleaning step was performed by leaving the fiber inside an injection port at the desorption temperature for 5 min. After this cleaning step, blank injections were performed and no carry-over was observed. The extraction efficiency of the prepared pPIL fiber coating was compared with the non-porous (npPIL) analogue and also with the commercial fiber DVB/CAR/PDMS (fused silica fiber coating, cross-linked with 50/30 µm divinylbenzene/carboxen™/polydimethylsiloxane StableFlex™ (1 cm, Sigma-Aldrich, Bellefonte, PA, USA)). This commercial fiber was selected due to its wide range capacity of sorbing compounds with different physicochemical properties^41^, being carboxen (CAR) responsible for imparting the fiber with pores. The first aspect to keep in mind is that while in the commercial fiber the non-polar porous structures are mixed within PDMS, the prepared pPIL-SPME fiber contains pores within the polymer, which, due to the hieratical architectural design of porous PILs, leads to the possibility of tuning interactions between analyte and extraction phase, owing to the type of extraction mechanisms involved (adsorption versus absorption and also simultaneous adsorption and absorption).

**Figure 8:**
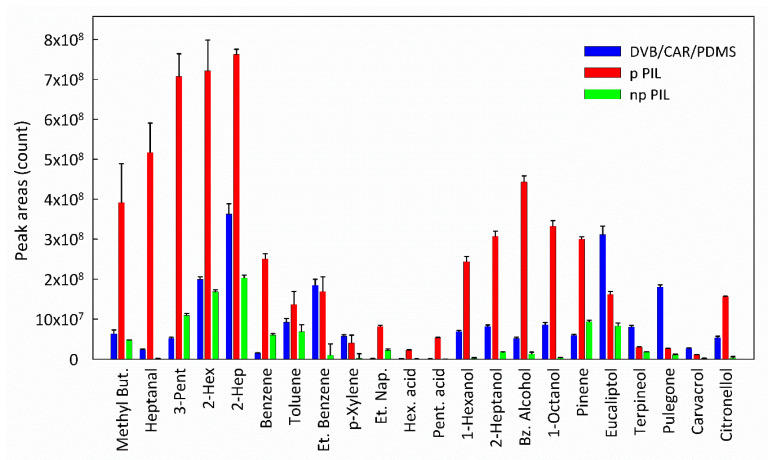
Extraction efficiency of the two PIL fibers and the commercial DVB/CAR/PDMS fiber using the prepared standard solution. Error bars show the standard deviation.

After the extraction step, each fiber was manually introduced into the injector port of a GC × GC–ToFMS system (details in “2.2 Experimental Methods”). Usually, for HS extractions, high temperature favors mass transfer [37]. Nevertheless, preliminary optimization experiments allowed the selection of a milder extraction temperature of 40 °C and a 30 min extraction time. Figure 8 presents the extraction efficiency of the three fibers under study.

The benefit of using two-dimensional gas chromatography is the capability of resolving overlapped signals, leading to short analysis times, while simultaneously detecting complex mixtures of analytes from different families. The main aspects readily noticed in Figure 8 are the superior extraction efficiency of the pPIL fiber over the commercial DVB/CAR/PDMS fiber, the wide sorption and high sensitivity of both PIL fibers, even taking into account the larger thickness of the commercial fiber, 85 µm [38] versus 20 µm of the PIL fibers, and the nature of the coating (liquid/porous versus solid (PIL)) [39]. The similar extraction profile of both PIL fibers is also noticed.

The presence of pores obtained using the interpolyelectrolyte complexation method resulted in much higher extraction efficiencies, especially for polar analytes with higher vapor pressures, such as in the case of 2-methylbutanal, heptanal, 3-pentanone, 2-hexanone or 2-heptanone. These analytes quickly occupy the pores, where adsorption and absorption processes take place, leading to better fiber selectivity. Since the used PIL presents rich hydrogen bond sites, polar analytes capable of undergoing hydrogen bonding were better extracted.

Nonetheless, the aromatic nature of the imidazolium cation leads to possible cation–π interactions. Therefore, excellent extraction capability towards benzene, benzyl alcohol and ethylnaphtalene, for example, was also achieved. These results clearly show the relevance of the proposed approach, which combines enhanced mass transfer processes, achieved through the presence of pores, with the capacity to promote specific interactions between PILs and solutes, due to the use of PILs as highly structure-tunable materials. It should be mentioned that the prepared fibers were used without loss of repeatability in more than 40 extraction cycles.

## 4. Conclusions

In summary, this work unveils the potential advantages of using porous PIL SPME fibers. It was clearly shown, by comparison of the extraction efficiency of the prepared pPIL SPME fiber with the nonporous PIL analogue, that porosity plays a crucial role when using PIL-based SPME fibers. Additionally, the effect of having polar porous materials can be unequivocally appreciated through the comparison of the extraction efficiencies of the as-prepared pPIL SPME fiber with the most commonly used commercial fibers, where a non-polar porous material is used. The potential of porous PIL fibers unleashed here is easily accessible through the versatile chemistry of PILs, by changing cations, anions and pendant groups. This is especially relevant since commercial fibers use a hydrophobic porous material, and with this approach the hydrophobicity of the PIL material can be tuned, further extending SPME applicability. These specific fibers can be especially important in the extraction of aromatic compounds or polar volatile compounds from complex matrices, namely, to respond to current challenges, such as to control the level of aromatic hydrocarbons contamination in food or environmental samples. In addition, polar volatiles extraction may be useful for metabolomics studies, revealing the perturbations in several pathways, such as lipid peroxidation or amino acids degradation.

## References

[1] Arthur C.L., Pawliszyn J. (1990). Solid phase microextraction with thermal desorption using fused silica optical fibers. Anal. Chem..

[2] Patinha D.J.D., Silvestre A.J.D., Marrucho I.M. (2019). Poly(ionic liquids) in solid phase microextraction: Recent advances and perspectives. Prog. Polym. Sci..

[3] Akiyama M., Murakami K., Ohtani N., Iwatsuki K., Sotoyama K., Wada A., Tokuno K., Iwabuchi H., Tanaka K. (2003). Analysis of volatile compounds released during the grinding of roasted coffee beans using solid-phase microextraction. J. Agric. Food Chem..

[4] Helin A., Rönkkö T., Parshintsev J., Hartonen K., Schilling B., Läubli T., Riekkola M.-L. (2015). Solid phase microextraction Arrow for the sampling of volatile amines in wastewater and atmosphere. J. Chromatogr. A.

[5] Matin A.A., Biparva P., Gheshlaghi M. (2014). Gas chromatographic determination of polycyclic aromatic hydrocarbons in water and smoked rice samples after solid-phase microextraction using multiwalled carbon nanotube loaded hollow fiber. J. Chromatogr. A.

[6] Flores G., Del Castillo M.L.R., Blanch G.P., Herraiz M. (2006). Detection of the adulteration of olive oils by solid phase microextraction and multidimensional gas chromatography. Food Chem..

[7] Bessonneau V., Zhan Y., De Lannoy I.A.M., Saldivia V., Pawliszyn J. (2015). In vivo solid-phase microextraction liquid chromatography-tandem mass spectrometry for monitoring blood eicosanoids time profile after lipopolysaccharide-induced inflammation in Sprague-Dawley rats. J. Chromatogr. A.

[8] Piri-Moghadam H., Alam M.N., Pawliszyn J. (2017). Review of geometries and coating materials in solid phase microextraction: Opportunities, limitations, and future perspectives. Anal. Chim. Acta.

[9] Souza Silva E.A., Pawliszyn J. (2012). Optimization of fiber coating structure enables direct immersion solid phase microextraction and high-throughput determination of complex samples. Anal. Chem..

[10] Naccarato A., Gionfriddo E., Elliani R., Pawliszyn J., Sindona G., Tagarelli A. (2018). Investigating the robustness and extraction performance of a matrix-compatible solid-phase microextraction coating in human urine and its application to assess 2–6-ring polycyclic aromatic hydrocarbons using GC–MS/MS. J. Sep. Sci..

[11] Hou X., Wang L., Guo Y. (2017). Recent developments in solid-phase microextraction coatings for Environmental and biological analysis. Chem. Lett..

[12] Feng J., Qiu H., Liu X., Jiang S., Feng J. (2013). The development of solid-phase microextraction fibers with metal wires as supporting substrates. TrACs Trends Anal. Chem..

[13] Yuan J., Mecerreyes D., Antonietti M. (2013). Poly(ionic liquid)s: An update. Prog. Polym. Sci..

[14] Mecerreyes D. (2011). Polymeric ionic liquids: Broadening the properties and applications of polyelectrolytes. Prog. Polym. Sci..

[15] Sun J.-K., Kochovski Z., Zhang W.-Y., Kirmse H., Lu Y., Antonietti M., Yuan J. (2017). General synthetic route toward highly dispersed metal clusters enabled by poly(ionic liquid)s. J. Am. Chem. Soc..

[16] Zhao F., Meng Y., Anderson J.L. (2008). Polymeric ionic liquids as selective coatings for the extraction of esters using solid-phase microextraction. J. Chromatogr. A.

[17] Young J.A., Zhang C., Devasurendra A.M., Tillekeratne L.M.V., Anderson J.L., Kirchhoff J.R. (2016). Conductive polymeric ionic liquids for electroanalysis and solid-phase microextraction. Anal. Chim. Acta.

[18] Patinha D.J.S., Tomé L.C., Isik M., Mecerreyes D., Silvestre A.J.D., Marrucho I.M. (2017). Expanding the applicability of poly(Ionic Liquids) in solid phase microextraction: Pyrrolidinium coatings. Materials.

[19] Patinha D.J.S., Pothanagandhi N., Vijayakrishna K., Silvestre A.J.D., Marrucho I.M. (2018). Layer-by-layer coated imidazolium–Styrene copolymers fibers for improved headspace-solid phase microextraction analysis of aromatic compounds. React. Funct. Polym..

[20] Yu H., Ho T.D., Anderson J.L. (2013). Ionic liquid and polymeric ionic liquid coatings in solid-phase microextraction. TrAC Trends Anal. Chem..

[21] Mei M., Huang X., Chen L. (2019). Recent development and applications of poly (ionic liquid)s in microextraction techniques. TrAC Trends Anal. Chem..

[22] Feng J., Sun M., Xu L., Wang S., Liu X., Jiang S. (2012). Novel double-confined polymeric ionic liquids as sorbents for solid-phase microextraction with enhanced stability and durability in high-ionic-strength solution. J. Chromatogr. A.

[23] Pang L., Liu J.F. (2012). Development of a solid-phase microextraction fiber by chemical binding of polymeric ionic liquid on a silica coated stainless steel wire. J. Chromatogr. A.

[24] Zhang Y., Wang X., Lin C., Fang G., Wang S. (2012). A novel SPME fiber chemically linked with 1-vinyl-3-hexadecylimidazolium hexafluorophosphate ionic liquid coupled with GC for the simultaneous determination of pyrethroids in vegetables. Chromatographia.

[25] Trujillo-Rodríguez M.J., Yu H., Cole W.T.S., Ho T.D., Pino V., Anderson J.L., Afonso A.M. (2014). Polymeric ionic liquid coatings versus commercial solid-phase microextraction coatings for the determination of volatile compounds in cheeses. Talanta.

[26] Feng J., Sun M., Li L., Wang X., Duan H., Luo C. (2014). Multiwalled carbon nanotubes-doped polymeric ionic liquids coating for multiple headspace solid-phase microextraction. Talanta.

[27] Mei M., Yu J., Huang X., Li H., Lin L., Yuan D. (2015). Monitoring of selected estrogen mimics in complicated samples using polymeric ionic liquid-based multiple monolithic fiber solid-phase microextraction combined with high-performance liquid chromatography. J. Chromatogr. A.

[28] Wu M., Wang L., Zeng B., Zhao F. (2016). Ionic liquid polymer functionalized carbon nanotubes-doped poly(3,4-ethylenedioxythiophene) for highly-efficient solid-phase microextraction of carbamate pesticides. J. Chromatogr. A.

[29] Lin H., Zhang S., Sun J.-K., Antonietti M., Yuan J. (2020). Poly(ionic liquid)s with engineered nanopores for energy and environmental applications. Polymer.

[30] Zhao Q., Yin M., Zhang A.P., Prescher S., Antonietti M., Yuan J. (2013). Hierarchically structured nanoporous poly(ionic liquid) membranes: Facile preparation and application in fiber-optic pH sensing. J. Am. Chem. Soc..

[31] Zhao Q., Fellinger T.-P., Antonietti M., Yuan J. (2013). A novel polymeric precursor for micro/mesoporous nitrogen-doped carbons. J. Mater. Chem. A.

[32] Levkin P.A., Svec F., Fréchet J.M.J. (2009). Porous polymer coatings: A versatile approach to superhydrophobic surfaces. Adv. Funct. Mater..

[33] Escalé P., Rubatat L., Billon L., Save M. (2012). Recent advances in honeycomb-structured porous polymer films prepared via breath figures. Eur. Polym. J..

[34] Li J., Zhang Y. (2007). Porous polymer films with size-tunable surface pores. Chem. Mater..

[35] Álvarez-Fernández A., Valdés-Bango F., Losada-Ambrinos R., Martín J.I., Vélez M., Alameda J.M., Alonso F.J.G. (2018). Polymer porous thin films obtained by direct spin coating. Polym. Int..

[36] Wang H., Min S., Ma C., Liu Z., Zhang W., Wang Q., Li D., Li Y., Turner S., Han Y. (2017). Synthesis of single-crystal-like nanoporous carbon membranes and their application in overall water splitting. Nat. Commun..

[37] Feng J., Sun M., Wang X., Liu X., Jiang S. (2012). Ionic liquids-based crosslinked copolymer sorbents for headspace solid-phase microextraction of polar alcohols. J. Chromatogr. A.

[38] Silva C., Cavaco C., Perestrelo R., Pereira J., Câmara J.S. (2014). Microextraction by packed Sorbent (MEPS) and solid-phase microextraction (SPME) as sample preparation procedures for the metabolomic profiling of urine. Metabolites.

[39] Jiang R., Pawliszyn J. (2012). Thin-film microextraction offers another geometry for solid-phase microextraction. TrAC Trends Anal. Chem..

